# Prehospital fentanyl administration by ambulance personnel

**DOI:** 10.1186/1757-7241-21-S2-A42

**Published:** 2013-09-09

**Authors:** KF Christensen, L Nikolajsen, H Kirkegaard, EF Christensen

**Affiliations:** 1 Dept. of Anesthesiology, Aarhus University Hospital; 2 Research Center for Emergency Medicine, University of Aarhus; 3 Research Dept., Prehospital Emergency Medical Services, Central Denmark Region Aarhus, Denmark

## Background

Acute pain is one of the most common problems faced in prehospital emergency medicine. Sufficient prehospital pain therapy reduces psychological and emotional stress. Other clinical benefits include optimized conditions for patient transport, increased patient satisfaction and a better chance of timely and proper analgesia at the emergency department. Unfortunately, undertreatment of acute pain (oligoanalgesia) is common. Oligoanalgesia is associated with following factors:

• Variable clinical experience

• Concern for masking illness or injury

• Focus on other clinical symptoms

• Poor education in pain management and insufficient compliance with pain management protocols

• Fear of inducing adverse effects

• Lack of follow-up after initial pain therapy administration

## Methods

One approach to minimize oligoanalgesia is to increase the use of fentanyl in the prehospital environment. Fentanyl is an opioid with rapid-acting properties and short time of action allowing safe titration and few side effects.

In order to optimize prehospital pain management all ambulance personnel in Central Denmark Region have been taught how to administer fentanyl in specific clinical situations and under certain circumstances. We wish to present the preliminary results from a 3-month period in which rescuers working for one of the two ambulance companies operating in the region, Responce and Falck, were allowed to administer fentanyl.

## Results

A total of 204 patients were treated with fentanyl by ambulance personnel over a period of 3 month. About one half had some kind of injury (n=114) and the remainder experienced pain due to acute coronary syndrome (n=52), abdominal pain (n=18) or other clinical conditions (n=20). None of the patients experienced side effects. Antidote was not required in any of the cases.

## Conclusion

The administration of fentanyl by ambulance personnel seems to be safe. Future studies will further evaluate pain and the safety and effectiveness of fentanyl administered by ambulance personnel in Central Denmark Region.

